# Conscious awareness modulates processing speed in the redundant signal effect

**DOI:** 10.1007/s00221-020-06008-1

**Published:** 2021-04-17

**Authors:** Anna Matilda Helena Cederblad, Aleksandar Visokomogilski, Søren K. Andersen, Mary-Joan MacLeod, Arash Sahraie

**Affiliations:** 1 School of Psychology, University of Aberdeen, Aberdeen, UK; 2 Institute of Medical Sciences, University of Aberdeen, Aberdeen, UK

**Keywords:** Redundant Signal Effect, Visual awareness, Continuous flash suppression

## Abstract

Evidence for the influence of unaware signals on behaviour has been reported in both patient groups and healthy observers using the Redundant Signal Effect (RSE). The RSE refers to faster manual reaction times to the onset of multiple simultaneously presented target than those to a single stimulus. These findings are robust and apply to unimodal and multi-modal sensory inputs. A number of studies on neurologically impaired cases have demonstrated that RSE can be found even in the absence of conscious experience of the redundant signals. Here, we investigated behavioural changes associated with awareness in healthy observers by using Continuous Flash Suppression to render observers unaware of redundant targets. Across three experiments, we found an association between reaction times to the onset of a consciously perceived target and the reported level of visual awareness of the redundant target, with higher awareness being associated with faster reaction times. However, in the absence of any awareness of the redundant target, we found no evidence for speeded reaction times and even weak evidence for an inhibitory effect (slowing down of reaction times) on response to the seen target. These findings reveal marked differences between healthy observers and blindsight patients in how aware and unaware information from different locations is integrated in the RSE.

## Introduction

Visual awareness has in the past proven not to be a prerequisite for visual information to be processed. Evidence of this has been found in healthy observers (e.g., Hesselmann et al. 2011; Hurme et al. 2017; Robichaud and Stelmach 2003) as well as in clinical populations (Pöppel et al. 1973; Weiskrantz et al. 1974). Unconscious vision, or a lack of visual awareness in parts of the visual field in clinical populations is often associated with lesions of the visual pathways. Some patients with cortical lesions in early visual areas may retain some residual capacity to process visual information restricted to their blind field, even when they are not consciously aware of its presentation (Pöppel et al. 1973; Riddoch 1917; Weiskrantz et al. 1974). This is known as blindsight, and is well established in a range of paradigms, including localisation (Weiskrantz et al. 1974), emotion discrimination (Morris et al. 2001, for a review see Celeghin et al. 2015a), navigation (de Gelder et al. 2008) and speeded processing (Marzi et al. 1986). However, the field of studying unconscious vision in healthy observers has not yielded entirely consistent findings (Kolb and Braun 1995; Morgan et al. 1997). When studying unconscious vision, or blindsight-like performance in healthy observers there are two aspects one must consider. Firstly, one must decide how to record ratings of subjective experience, such as clarity of visual experience (e.g., Aller et al. 2015), confidence in the response given (e.g., Robichaud and Stelmach 2003), or to combine and examine the relationship between both objective and subjective measures (Hesselmann et al. 2011). Secondly, the paradigm chosen must lead to comparable findings. In order to do this, one must apply a method which can make information fall outside of conscious awareness in normal observers whilst maintaining an above chance level of accuracy. This has been attempted by the use of a range of methods including reducing the signal to noise ratio for some targets by showing near- or sub-threshold stimuli (Savazzi and Marzi 2002, 2008) or by using Transcranial Magnetic Stimulation (TMS) to momentarily disrupt neuronal processing at specific cortical regions (Boyer et al. 2005; Hurme et al. 2017). Another example of a method which has shown blindsight-like behaviour in healthy observers is the use of binocular rivalry (Baker and Cass 2013; Kolb and Braun 1995) although these reports of blindsight-like performance with above chance accuracy outside of conscious awareness (similar to blindsight patients) has not been readily replicated in healthy adults (Morgan et al. 1997; Robichaud and Stelmach 2003). Continuous Flash Suppression (CFS) has been offered as an alternative method for simulating a blindsight-like behaviour in healthy observers (Hesselmann et al. 2011) as it can mask part of the visual field while information can be presented underneath the CFS-mask (Tsuchiya and Koch 2005). Using a mirror set-up, the information input to each eye is separated such that one eye (often the non-dominant eye) is presented with the target stimulus and a dynamic random noise pattern is presented to the remaining eye (Tsuchiya and Koch 2005). At low target stimulus contrasts, the dynamic noise patterns dominate and suppress the awareness of the target stimulus. Increasing the stimulus target contrast will allow it to break through and be detected. This measure, termed breaking CFS (b-CFS), or the priming effect of a CFS-masked stimulus on detection or processing of a subsequent visual targets are used to investigate the unconscious processing of a wide range of stimuli such as facial emotional expressions (Yang et al. 2014), selective object processing (Kaunitz et al. 2011), and social influences and preferential processing of self-relevant information (Macrae et al. 2017). However, evidence for unconscious processing of supressed stimuli that require integration across basic features to process objects as whole have been inconsistent. The earlier reports of intact unconscious object processing along the dorsal pathway as opposed to impaired processing along the ventral route (Fang and He 2005) have not been replicated (Hesselmann et al. 2018) with findings being more consistent with proposal for object fractionations under CFS (Moors et al. 2017) with unconscious processing being limited to basic visual features (for a review, please see Ludwig and Hesselmann 2015).

Detection of supra-threshold stimuli across all modalities, despite being perceived as effortless, can lead to a range of reaction times. The variance in reaction time is often attributed to a combination of neuronal states during encoding of signals (e.g., the amount of noise or signal strength), decisional processes (e.g., evidence accumulation, decisional threshold or bias) and non-decisional components (e.g., variations in motor preparation and execution time) (Luce 1986; Pitts et al. 2014a, b; Pitts et al. 2014a, b). When combining evidence from multiple signals within or across modalities (such as synchronised visual and auditory events), the reaction times to combined stimuli are faster than those to each component separately (Aller et al. 2015; Todd 1912). The faster response to multiple targets compared to single targets is known as The Redundant Signal Effect (RSE) where the speeded response to multiple targets is termed redundancy gain (Todd 1912). Visual stimulation in an RSE paradigm is ideally suited to study unconscious vision in clinical blindsight as stimuli can be placed within, or outside of the blind field of hemianopic patients. The influence of unconscious processing of one signal on another consciously observed events can be investigated by requiring the patients to respond to a seen target that may or may not be accompanied by a second target presented within their visual field defect. In a limited number of cases, faster responses have been reported if the seen target was accompanied by an unconsciously processed target (Leh et al. 2006). One study in a small group of hemispherectomised patients demonstrated that RSE only occurred if there was an intact projection from the midbrain of the lesioned side, or more specifically from the Superior Colliculus in the damaged hemisphere to the intact hemisphere (Tomaiuolo et al. 1997). The contribution of Superior Colliculus to the RSE has been confirmed in patients with damaged midbrain (van Koningsbruggen et al. 2017). Thus, there are several examples of support for RSE outside of awareness in clinical blindsight; however, it is unclear how awareness could affect the performance of healthy observers in an RSE paradigm.

When RSE has been applied in studies where awareness or clarity of experience is of relevance but not explicitly recorded in healthy subjects, some assumptions tend to be made. One is that awareness of supra-threshold stimuli and a lack of awareness of sub-threshold stimuli is implicitly assumed throughout the study. Savazzi and Marzi (2002, 2008), for example, used subthreshold stimuli paired with supra-threshold stimuli and showed that detection of supra-threshold stimuli can benefit from a simultaneous display of a subthreshold stimuli. However, the participants’ subjective experience of the subthreshold targets was not recorded on a trial by trial basis, so it is not known whether the participants were indeed unaware of the subthreshold target in all trials. As CFS has also been proposed as a suitable analogue to blindsight in healthy observers (Hesselmann et al. 2011), it is of interest to examine what would happen if an RSE paradigm was applied under CFS. Specifically, whether evidence of facilitation in processing speed with faster reaction times associated with unconscious processing can be demonstrated similar to those reported in clinical blindsight (Georgy et al. 2016; Leh et al. 2006; Marzi et al. 1986; Tomaiuolo et al. 1997).

The Superior Colliculus has been pointed out as a crucial region for RSE to occur in blindsight in hemianopic patients, including hemispherectomy patients (Leh et al. 2006; Tomaiuolo et al. 1997). The Superior Colliculus has also been suggested to be activated by gestalt-like stimuli as is supported by findings in hemianopic patients with faster reaction times in an RSE paradigm for stimulation to the blind field with higher number of visual targets and when the targets were placed in a configuration of recognisable shapes as opposed to random positions (Celeghin et al. 2015b; Georgy et al. 2016). This raises the question as to whether the stimulus configuration could have a similar effect in healthy observers when CFS is used to simulate blindsight.

Here we report on three experiments in which we investigated how conscious and unconscious vision is associated with changes in behaviour in healthy observers. We investigated the effect of awareness on the speed of processing (manual reaction times) of double and single target displays when the redundant targets were placed beneath a CFS mask, sometimes rendering the observer unaware of its presentation. Participants reported their subjective experience of the clarity of the redundant target using the Perceptual Awareness Scale (PAS) (Ramsøy and Overgaard 2004) on every trial. The first experiment had a classic RSE paradigm. In the second and third experiments we introduced uncertainty on the spatial location of the masked target confined to either contralateral visual field (Exp 2) or in either hemifield (Exp 3) to the unmasked target. Across the three experiments we found that awareness of all targets in a multi-target display was crucial for RSE to occur in healthy observers.

## General methods

In all three experiments, participants were informed of the task instructions and consented to take part. Ethical approval was granted for all studies by the School of Psychology Ethics Committee. The participants were recruited through the University of Aberdeen’s online notice board and the University of Aberdeen School of Psychology student participant scheme. The participants were either paid (£8 for experiments 1 and 2, £10 for experiment 3) or awarded course credit for their participation.

The experiments were developed in E-Prime (E-Prime 2.0, Psychology Software Tools Inc. USA) and the stimuli were generated in GIMP (version 2.8.22 GNU Image Manipulation Program). The visual targets of all experiments were circles (1.09° diameter) that were placed 3.5° away from the centre fixation. The visual targets were presented for 100 ms in each trial. The left and right eye images were presented on separate but identical monitors with each image having a fixed border (0.27°) that matched in both images. The combination of fixed border and fixation cross allowed for comfortable and stable fusion of the two images using a mirror set-up. The two LCD monitors used in the experiments (EliteDisplay E202, Hewlett-Packard, USA) had identical background grey luminance (40 cd/m^2^) and all luminance measurements for targets and backgrounds were carried out using a Luminance meter (LS-100, Minolta, Japan).

**Fig. 1:**
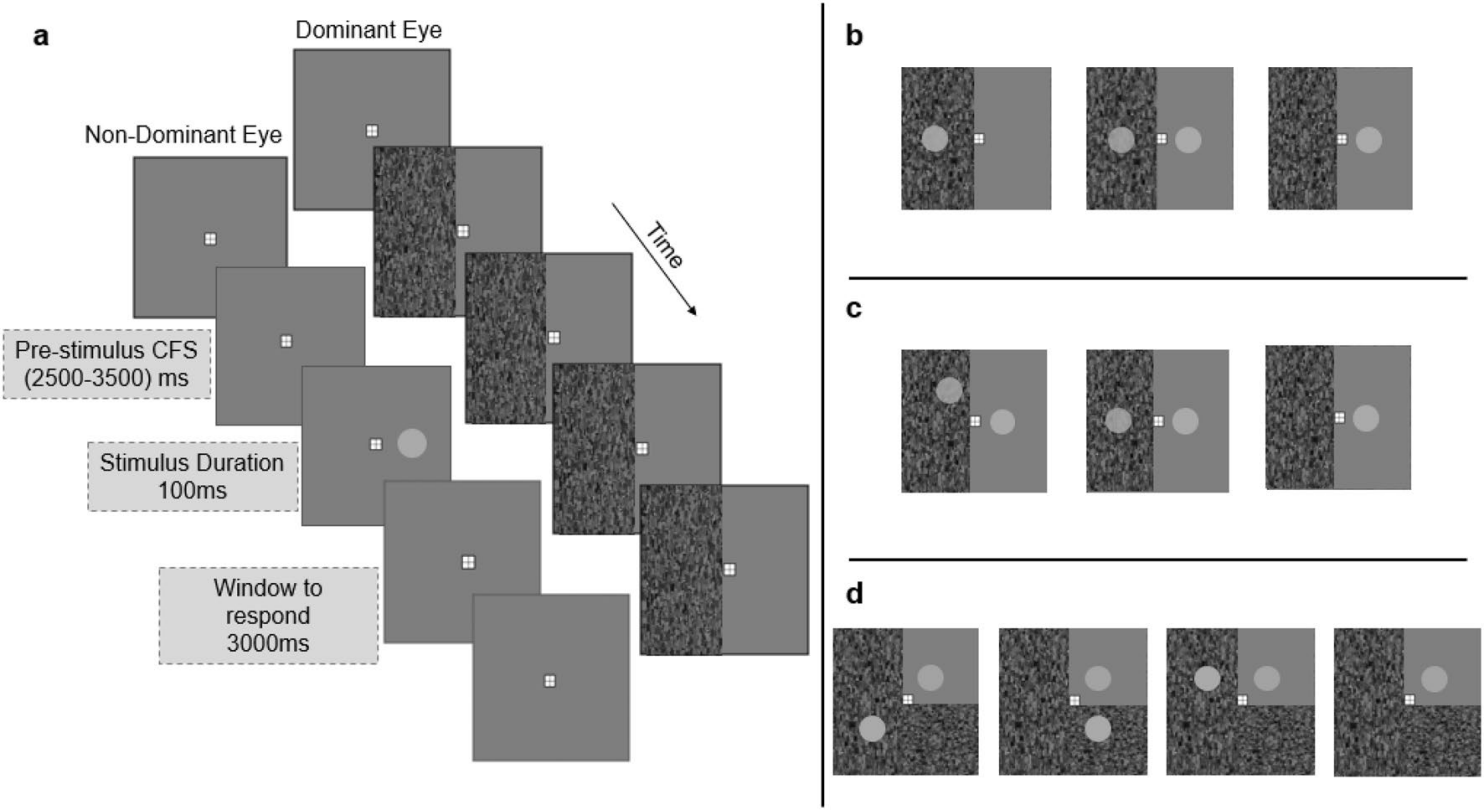
**a** Schematic representation of the time-course of a trial for a Single unmasked target. Schematic displays of the spatial locations of the visual targets of experiment 1(**b**), experiment 2(**c**) and experiment 3(**d**)

The dominant eye of each participant was determined using a “hole in the hand test” based on the Miles test (Miles 1930). A dynamic achromatic Mondrian (10 Hz) was applied to a portion of the visual field in their dominant eye, the extent of which is specified for each experiment in Figs. 1. In all experiments head movements were minimised using a head/chin rest set at an optical viewing distance of 42 cm from the display monitors.

### Threshold measurements

For each participant, the target contrast necessary for detection of a circular target (1.09° diameter) presented for 100 ms (ISI = 500 ms) was determined using a method of limits. For experiment 1 the targets were located 3.5° to either the left- or right-hand side of the centred fixation. With each consecutive presentation, the target contrast was increased by 1% Michelson contrast. The participant was instructed to keep their gaze on a central fixation cross and respond as fast as they could by pressing the space bar on a keyboard when they perceived the target. The procedure was repeated 20 times for both masked (under CFS) and unmasked target presentations and the mean and standard deviation for the threshold measurements were calculated. For each participant, the contrast of the masked target stimuli was set to two standard deviations below their mean threshold for masked conditions. The threshold for detection of unmasked target was only measured in experiment 1 but was not used to set the stimuli contrast for the experiment. The above procedure was followed for all three experiments. The locations of the stimuli were matched to those reported in experiments 2 and 3 (see Fig. 1c, d).

### Reaction time measurements

The reaction time to target onset was the main dependent variable in all three experiments. Responses that were faster than 200 ms were excluded from the analysis as these were considered to be anticipatory. Trials in which no response was made (missed trials) were also excluded. Some participants were excluded based on their overall performance. The outlier removal was based on excessively slow reaction times and non-compliance with instructions (median reaction time for a condition 2SD slower than the group mean of medians). The number of these occurrences are reported for each experiment.

### Subjective awareness rating

After each trial, the participants were asked to verbally report their level of subjective awareness of a target that they may or may not have seen in the masked field. Awareness ratings were verbally reported according to the 4-point Perceptual Awareness Scale (Ramsøy and Overgaard 2004). Participants were provided with the following written descriptions: 1—“No experience”, 2—“Brief glimpse”, 3—“Almost clear experience”, 4—“Clear experience”. The verbal responses were recorded via a keypress by the experimenter.

Due to the low target luminance contrasts applied, there were few responses in the upper PAS categories (3 and 4) across all experiments. Therefore, all responses that were indicative of any level of awareness of targets under the masked condition (PAS score > 1) were clustered into one category of “Aware” responses and all responses with a PAS score of 1 were categorised as “Unaware” in the analysis for each experiment separately. For each condition and experiment, the number and proportion of aware and unaware responses and missed trials are reported. The frequency of each PAS response is also reported for each experiment. In the Linear Mixed Effect Model analysis, where all the data from the three experiments is combined in one analysis, the PAS responses are entered in the four-point scale (see Fig. 6a, b).

### Statistical analysis

Multiple comparisons in the ANOVAs were corrected using the Bonferroni method. There were 20 participants in experiments 1 and 2, and 25 in experiment 3. Where the degrees of freedom are lower than 19 (Experiment 1 and 2) or 24 (Experiment 3), in t-tests of the results sections this is due to missing cases for that analysis.

## Experiment 1—RSE and unconscious vision in healthy observers

The objective of experiment 1 was to investigate how awareness of visual information can affect speed of processing, that is, whether healthy observers can show an RSE under unconscious vision. To assess this, we applied a typical RSE paradigm combined with a CFS mask covering half of the visual field of the observers.

### Participants

Twenty participants (16 female, age range 19–28, *M* = 23.55, SD = 3.1) were recruited, eight of whom were left-eye dominant, and two were left-handed. The above sample was obtained after two participants were excluded for not complying with the instructions, and one was excluded for being an outlier with an average reaction time that was slower than two standard deviation above the group mean.

### Procedure

In the first experiment we collected reaction time data on three experimental conditions, namely, a single stimulus presentation either under masked field (under CFS) or unmasked field or both fields. The participants’ task was to press a response key as soon as they saw any dot target appear as well as rate their awareness of targets presented in the masked field. One hundred trials were presented for each of the three stimulus configurations. Trials were presented in random order and grouped into four blocks of 75 trials. Half of the visual field of the dominant eye was covered with the CFS mask. A schematic representation of a trial and information on timing of events are shown in Fig. 1. The stimulus configurations in experiment 1 were one Double target (Double Contralateral), and two single target conditions. The single target conditions are referred to as the Single Masked (target presented in the CFS mask) and Single unmasked (target presented outside of the mask) condition. The target was displayed at a random time between 2000 and 3000 ms after the mask appeared. The mean contrast threshold of the masked field for experiment 1 was *M* = 12.52%, SD = 3.17, and the mean for the stimuli used was *M* = 6.65%, SD = 1.35.

### Results of experiment 1

The single masked condition was distinguished in that it had a high percentage of “no response” trials (in total *n* = 1032 trials, 51.6% of all trials for the condition. *M* = 51.6, SD = 30.72, range 5–99) compared to the Single unmasked (in total *n* = 52 trials, 2.6% of all trials for the condition. M = 2.6, SD = 3.14, range 0–10) and the Double Contralateral target conditions (in total *n* = 55 trials, 2.75% of all trials for the condition. *M* = 2.75, SD = 3.74, range 0–13). For each participant, median RTs for aware and unaware trials for each condition were calculated and group averages represent the mean of all participants’ median reaction times. There were 51 excluded anticipatory trials which represented 0.85% of all trials (reaction time faster than 200 ms). There was variation in the number of PAS responses per participant for the Double Contralateral target condition the average PAS 1 response was 42.8 (SD = 32.54, range 4–99), PAS 2 average was 35.4 (SD = 21.79, range 0–64), PAS 3 average was 15.25 (SD = 18.13, range 0–55), PAS 4 average was 3.8 (SD = 7.69, range 0–28). The pooled data for PAS responses 2–4 for the Double Contralateral target condition was on average 54.45 (SD = 34.25, range = 0–96). For the Single unmasked condition there was an average of 84.75 PAS 1 responses (SD = 17.72, range 50–100), PAS 2 average 11.65 (SD = 15.28, range 0–47), PAS 3 average 0.85 (SD = 2.08, range 0–9), PAS 4 average 0.15 (SD = 0.67, range 0–3) (see Fig. 1c). The pooled data for PAS responses 2–4 for the Single unmasked condition was on average 12.65 (SD = 16.41, range = 0–47).

The first analysis examined how stimulus configurations affected reaction times without accounting for subjective awareness ratings. A repeated measures ANOVA showed that there was a significant effect of stimulus configuration [*F*(1.006, 18.101) = 24.303, *p* < 0.001, *η*_p_^2^ = 0.574] (Greenhouse–Geisser corrected, uncorrected df = 2, 36). Reaction times in the Double and the Single unmasked conditions were not significantly different from one another [*t*(19) = − 0.447, *p* = 0.66]. The mean reaction time for the single masked condition (*M* = 867 ms, SD = 350) was almost twice as high as the Single unmasked (*M* = 486 ms, SD = 75) and the Double Contralateral target (*M* = 484 ms, SD = 83) conditions.

**Fig. 2:**
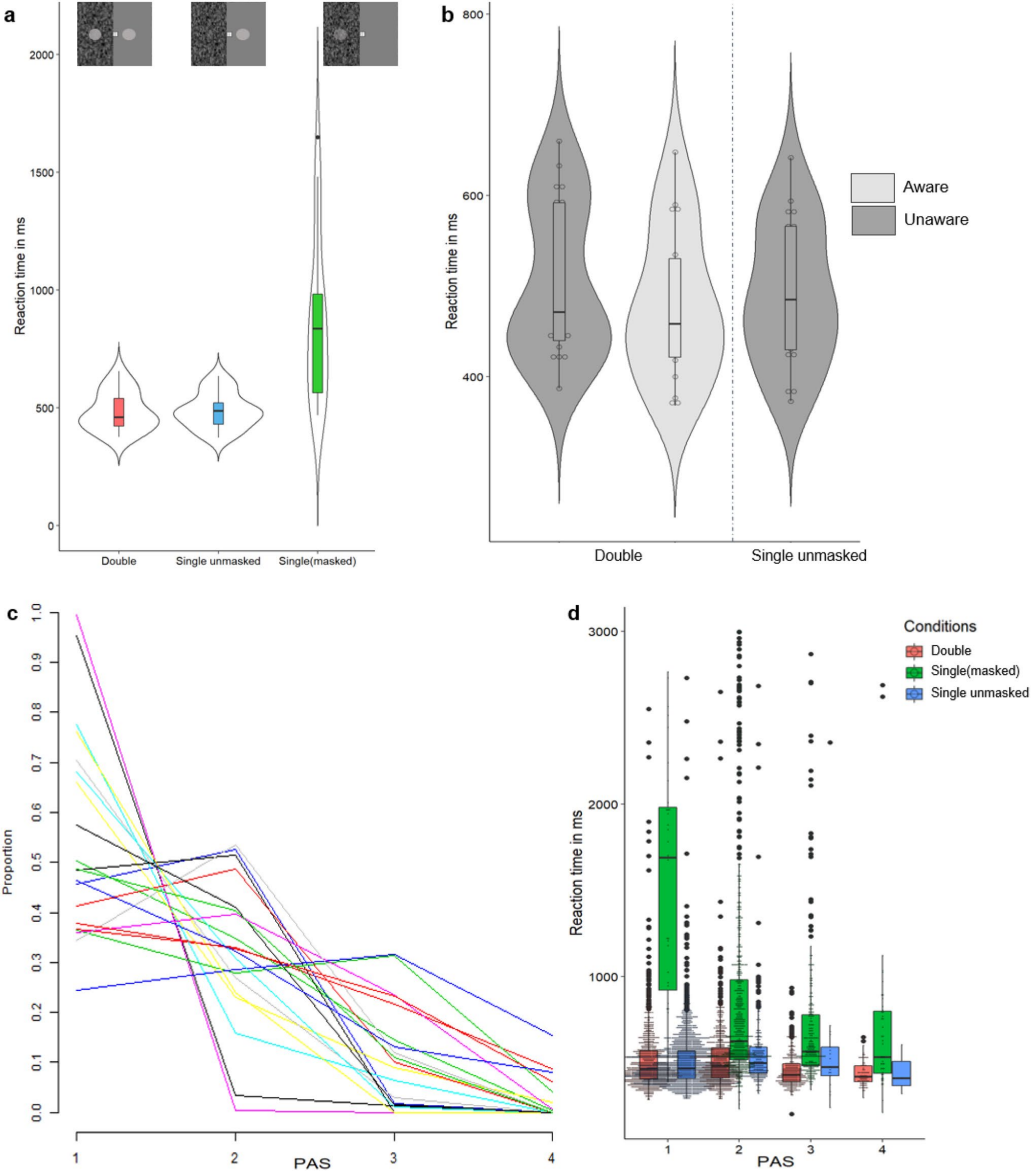
**a** Violin plot with boxplot of the reaction times split by condition. **b** Violin plots with box plots of the reaction times to the Double Contralateral condition split by Aware and Unaware trials, and the Single unmasked trials. Each dot represents the mean reaction time for each participant. **c** The proportion of PAS 1, 2, 3, and 4 responses per participant. Each participant of experiment 1 is represented by a line. **d** A boxplot of the reaction time per condition and available PAS response with the data by trial of experiment 1 overlaid as a dot plot. **c**, **d** were made in R with the package ggplot2 (Wickham 2016)

A second analysis was conducted to investigate differences in reaction time based on subjective awareness ratings for the masked redundant target for the Double Contralateral and Single unmasked target conditions (only including Single unmasked trials where participants accurately reported to be unaware of the absent, masked redundant target). For each participant, the Double Contralateral target condition was split into trials where the subject was aware (*n* = 1089 trials, 54.45%) versus unaware (*n* = 856 trials, 42.8%) of a target appearing in the masked visual field (2.75% missing trials in the Double Contralateral target condition). The reaction times for the Double Contralateral Aware (*M* = 478 ms, SD = 83) and Unaware (*M* = 506 ms, SD = 86) were compared to the reaction time for Single unmasked trials (*n* = 1695 trials, 84.75% excluding trials that participants reported awareness of a masked target where none was presented *n* = 253 trials, 12.65%, =; and missing trials 2.6%) (*M* = 492 ms, SD = 78). Double Aware trials were significantly faster than Single unmasked trials [*t*(17) = − 2.36, *p* = 0.03, Cohen’s *d* = 0.174]. Double Unaware trials were significantly slower than Single unmasked [t(19) = 2.132, *p* = 0.046, Cohen’s *d* = 0.171] (Fig. 2).

### Summary of experiment 1

Experiment 1 showed that for the RSE to occur in healthy observers, they need to be aware of the presentation of the redundant target. If an observer reports to be unaware of a redundant target, then this was associated with a slower response compared to when they were aware of the masked target. Thus, it seems that in order to benefit from speeded processing associated with the display of multiple targets, a healthy observer needs to be aware of all targets presented. This is not in agreement with findings from studies with hemianopia and hemispherectomy patients who still show an RSE even when targets are presented within their blind field (Leh et al. 2006; Marzi et al. 1986; Tomaiuolo et al. 1997). Indeed, the finding of a significant inhibitory effect of unaware target on the reaction times to the onset of an unmasked target is surprising. In experiment two we aimed to investigate the robustness of this finding.

## Experiment 2—uncertainty of the masked target location

Findings from experiment 1 indicated that an observer needs to be aware of all targets presented to show an RSE. Thus, the observers’ reported experience of seeing or not seeing a masked target was associated with differences in processing speed in a redundant target display. In the previous experiment, participants were asked to fixate on a fixation point and stimuli were presented at fixed locations. Some stimulus judgements relying on attentional allocation to stimuli within peripheral field of vision such as between hemisphere size judgement, have been shown to vary as a function of eccentricity and spatial uncertainty (Charles et al. 2007). To explore whether eccentricity or spatial location was a determining factor leading to speeded responses in redundant target condition, we varied the spatial location of the target in experiment 2.

### Participants

Twenty naïve participants (16 female, age range 18–28, *M* = 21.30, SD = 2.98) took part. Ten participants were left-eye dominant, and two were left-handed. The above sample was obtained after two participants were removed from analysis as they did not comply with the instructions and one was excluded as an outlier with an average reaction time slower than the group mean per condition plus two standard deviations.

### Procedure

Masked targets were presented in either one of two locations, one to the left and another along the 45° meridian from the centre fixation, both at an eccentricity of 3.5°. We have termed this configuration “Double Asymmetric” as the masked and unmasked targets were not presented in mirror symmetry with respect to the vertical meridian as was the case for “Double Contralateral” configuration (see Fig. 1c for a graphical representation of the three configurations). We removed the single masked target condition as it was redundant to the purpose of the current experiment, but the Single Unmasked condition was included. Similar to experiment 1, the threshold for detection of a target for both masked locations were obtained, but as these values for all participants were nearly identical, a single stimulus contrast was chosen for each participant as two standard deviations below the mean of threshold at both locations. The three conditions were presented for 100 trials each in a randomised order and in four blocks. On average, the experiment took 90 min to complete. The mean contrast threshold of the masked field for experiment 2 was *M* = 12.69%, SD = 2.92, and the mean for the stimuli used in experiment 2 was *M* = 5.95%, SD = 1.4.

### Results of experiment 2

The initial analysis tested for any differences between stimulus configurations without accounting for awareness. A repeated measures ANOVA showed that there was a significant main effect of stimulus configuration on reaction times [*F*(2,38) = 4.494, *p* = 0.018, *η*_p_^2^ = 0.191]. Pairwise comparisons showed that responses to the Single unmasked condition (*M* = 521 ms, SD = 116) were slower than to both the Double Contralateral [*M* = 507 ms, SD = 98, *t*(19) = -2.259, *p* = 0.036], and the Double Asymmetric [*M* = 506 ms, SD = 104, *t*(19) = − 2.594, *p* = 0.018), configurations. There were in total 2.3% (*n* = 138, average 6.9, range 0–40) missing and 0.93% (*n* = 56) anticipatory (below 200 ms) responses in experiment 2, which were excluded from the analysis.

The frequency of PAS responses per condition were as follows. For the Double Contralateral condition there were on average 32.45 PAS 1 responses (SD = 22.64, range 6–88), PAS 2 average 41.9 (SD = 23.59, range 7–86), PAS 3 average 15.9 (SD = 13.89, 0–41), PAS 4 average 6.8 (SD = 14.19, range 0–51). For the Asymmetric condition there was on average 40.7 PAS 1 responses (25.06, range 1–84), PAS 2 average 36.7 (SD = 19.22, range 8–83), PAS 3 average 14.25 (SD = 17.03, range 0–53), PAS 4 average 4.95 (SD = 11.42, range 0–50). The Single unmasked condition had an average PAS 1 response of 83.3 (SD = 16.27, range 45–99), PAS 2 average 12 (SD = 11.34, range 0–38), PAS 3 average 1.2 (SD = 2.67, range 0–10), PAS 4 average 0.05 (SD = 0.22, range 0–1).

Secondly, the data was split into aware and unaware trials for the Double Contralateral and Asymmetric configurations. The number of aware trials did not differ between these two stimulus configurations [*t*(19) = 1.891, *p* = 0.074]. A 2 × 2 Repeated Measures ANOVA with the factors, stimulus configuration (symmetric/asymmetric) and awareness (aware/unaware) was conducted. This analysis showed that there was a significant main effect of subjective awareness [*F*(1,19) = 12.149, *p* = 0.002, *η*_p_^2^ = 0.39], with faster responses for Double Aware (Double Contralateral *M* = 499 ms, SD = 96, Double Asymmetric *M* = 505 ms, SD = 107) compared to Double Unaware (Double Contralateral *M* = 534 ms, *SD* = 108, Double Asymmetric *M* = 526 ms, SD = 111) trials. There was no main effect of stimulus configuration [*F*(1,19) = 0.031, *p* = 0.861, *η*_p_^2^ = 0.002] and no interaction between stimulus configuration and awareness [*F*(1,19) = 2.032, *p* = 0.17, *η*_p_^2^ = 0.097].

**Fig. 3:**
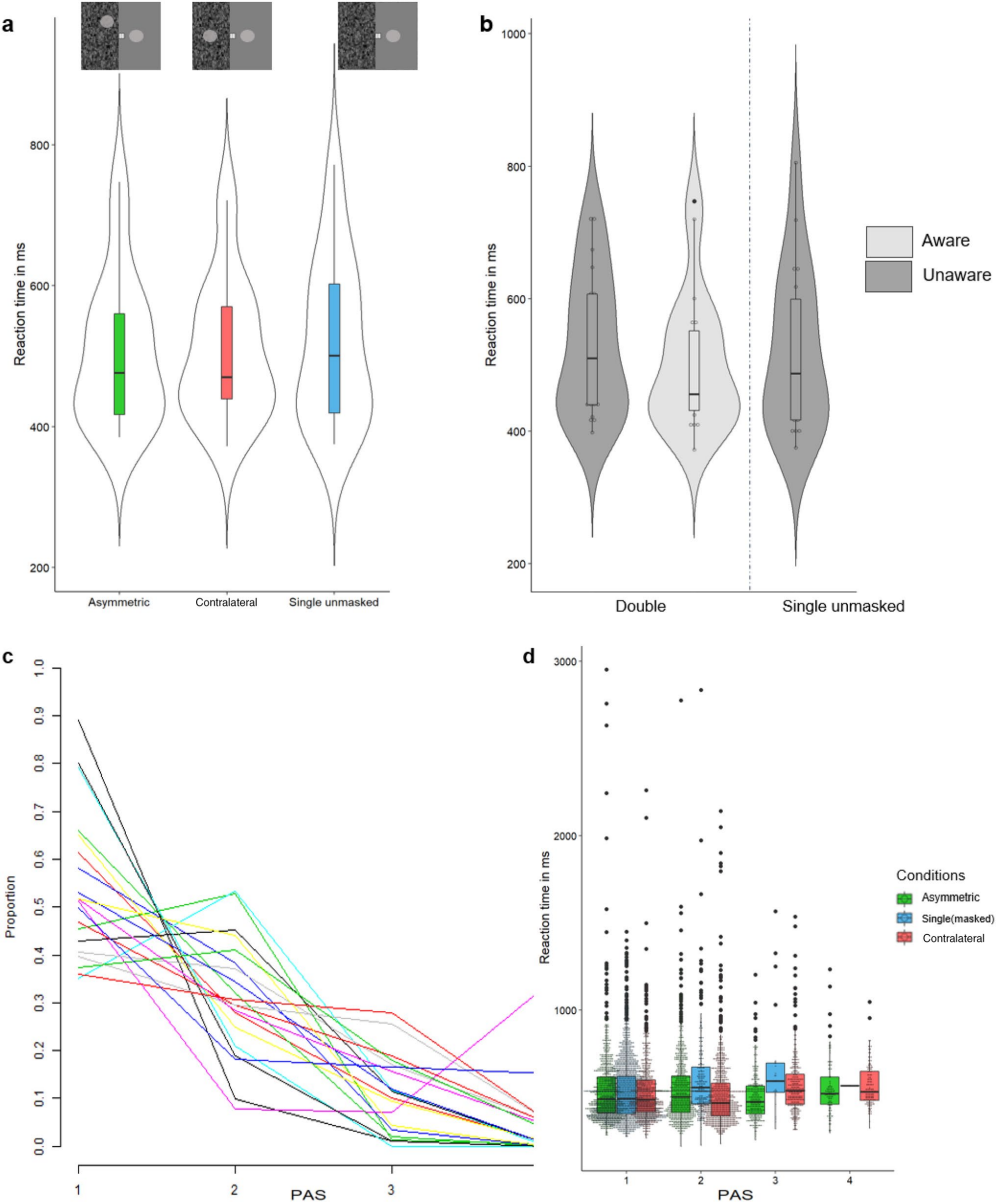
**a** Violin plot with boxplot of the reaction times split by condition. **b** Violin plots with box plots of the mean of the reaction times to the Double Asymmetric and Double Contralateral condition collapsed together and split by Aware and Unaware trials, and the Single unmasked condition. Each dot represents the mean for each participant. **c** The proportion of PAS 1, 2, 3, and 4 responses per participant. Each participant of experiment 2 is represented by a line. **d** A boxplot of the reaction time per condition and available PAS response with the data by trial of experiment 2 overlaid as a dot plot. **c**, **d** were made in R with the package ggplot2 (Wickham 2016)

Finally, we tested whether the RSE depended on subjective awareness. As the previous analysis did not reveal a main effect or interaction of stimulus configuration, we averaged reaction times across the Double Contralateral and Double Asymmetric conditions for aware (Double Contralateral: total *n* = 1292, average number of trials per participant = 64.6, range 11–94. Asymmetric: total *n* = 1118, average number of trials per participant = 55.9, range 14–99) and unaware (Symmetric: total *n* = 649, average number of trials per participant = 32.45, range 6–88. Asymmetric: total *n* = 814, average number of trials per participant = 40.7, range 1–84) trials separately, before comparing them against the Single unmasked condition (only including Single unmasked trials where participants accurately reported to be unaware of the absent, masked redundant target). Reaction times in the Double Aware trials (*M* = 501 ms, SD = 102) were significantly faster [*t*(19) = − 2.782, *p* = 0.012, Cohen’s *d* = 0.18] than in the Single unmasked trials (*M* = 521 ms, SD = 120, n = 1666, average number of trials per participant = 83.3, range 45–99.). Reaction times in the Double Unaware trials (*M* = 528 ms, SD = 106) did not differ significantly from the Single unmasked trials [*t*(19) = 1.322, *p* = 0.202, Cohen’s *d* = 0.06) (Fig. 3).

### Summary of experiment 2

The findings of experiment 2 replicated those in the previous experiment in that for the RSE to occur in healthy observers, they needed to be aware of all targets in a double target display. This was regardless of the location of the masked target, which showed that uncertainty of the location of the masked target did not influence reaction time or number of aware responses. However, the finding of an inhibitory effect of unaware masked targets in double target presentations was not replicated. In the face of two conflicting findings from the previous two experiments, we have reported below, on a third experiment to determine the relationship between RSE and awareness as well as any possible effect of stimulus configuration.

## Experiment 3—configuration of targets in a multi-target display

The third experiment was aimed at investigating the probable effects of the position of the masked target on awareness and reaction time. It has been suggested that the configuration of targets in a multi-target display can aid in speeding up processing of targets in the blind field of hemianopia patients (Celeghin et al. 2015b; Georgy et al. 2016). Experiment 1 and 2 have shown that awareness of targets is necessary for RSE to happen in healthy observers and experiment 2 showed that uncertainty of the location of the masked target did not change this outcome.

In almost all of the studies investigating the contribution of awareness to RSE in clinical populations, the target and redundant stimuli are shown symmetrically with some degree of variation in intact and impaired hemifields respectively (Celeghin et al. 2015b; Marzi et al. 1986; Tomaiuolo et al. 1997). The findings are then often discussed based on interhemispheric interactions at subcortical regions (Leh et al. 2006). In healthy observers between hemisphere object size judgments have been shown to be influenced by the symmetrical presentation of the stimuli with respect to vertical meridian (Charles et al. 2007). The CFS technique allows us to induce unaware stimuli in both hemifields in healthy observers, simulating a “blind” area within their visual field. Therefore, experiment 3 was aimed at determining if there was any difference in reaction time or number of aware responses at different spatial locations of the masked targets. The configuration of the redundant target conditions of experiment 3 all possessed some form of mirror symmetry with respect to the vertical meridian (Double Contralateral), horizontal meridian (Double Ipsilateral) and diagonal through the fixation point (Double Diagonal) (see Fig. 1d). These configurations can also establish the reliance of any putative inhibitory effects of unseen targets on cross-field symmetries.

### Participants

Twenty-five participants (21 female, age range 20–43, *M* = 26.16, SD = 5.6) were recruited to take part in the experiment. Sixteen participants were left-eye dominant, and four were left-handed. The above sample was obtained after three participants were excluded from the analysis due to not complying with instructions (almost always reported to be aware of the masked target, also for the Single unmasked condition which was an indicator that the participant was not doing the task as instructed) and two for being outliers with an median reaction time that was slower than the group mean per condition plus two standard deviations.

### Procedure

The experimental procedure of experiment 3 was similar to that of experiment 2 with the exception that in order to allow for the inclusion of Double Ipsilateral condition, the mask covered three quarters of the visual field (see Fig. 1d for graphical representation of the stimulus configurations). The participants’ task was again to press a key when they detected a target and then to report their awareness only of targets in the mask on a four-point scale. For each participant, threshold detections were obtained and averaged for all three masked locations and target luminance were set to two standard deviations below the average detection threshold. The conditions were presented in a randomised order in five blocks (in total 100 trials per condition). The mean contrast threshold of the masked field for experiment 3 was *M* = 10.85%, SD = 1.55, and the range for the stimuli used in experiment 3 was *M* = 6.68%, SD = 1.75.

### Results of experiment 3

The initial analysis was conducted to test for any differences between stimulus configurations without accounting for awareness. A repeated measures ANOVA showed that there was a significant effect of stimulus configuration [*F*(3,72) = 4.649, *p* = 0.005, *η*_p_^2^ = 0.162] where the configurations were Single unmasked, Double Contralateral, Double Ipsilateral, and Double Diagonal. This was followed by paired samples t-test which revealed that RTs to the Single unmasked condition were significantly slower (*M* = 478 ms, SD = 68) than to the Diagonal condition [*M* = 463 ms, SD = 61; *t*(24) = − 2.882, *p* = 0.008] and also significantly slower than the Contralateral configuration [*M* = 468 ms, SD = 58; *t*(24) = − 2.227, *p* = 0.036]. There was no significant difference in reaction times between the Single unmasked and the Ipsilateral configuration [*M* = 471 ms, SD = 64; *t*(24) = − 1.66, *p* = 0.11]. There were in total 1.68% (*n* = 168, average 6.72, range 0–48) missing and 1.75% (*n* = 175) anticipatory (below 200 ms) trials in experiment 3.

The frequency of PAS responses by condition was as follows. Diagonal average PAS 1 39 (SD = 28.1, range 2–88), PAS 2 average 36.8 (SD = 22.95, 0–82), PAS 3 average 12.96 (SD = 14.03, 0–43), PAS 4 average 7.96 (SD = 19.08, range 0–86). For the Ipsilateral there was an average of 38.56 PAS 1 responses (SD = 24.08, 8–87), PAS 2 average 44 (SD = 23.11, range 5–84), PAS 3 average 10.16 (SD = 11.64, range 0–38), PAS 4 average 3.72 (SD = 13.56, range 0–68). For the Contralateral condition there was an average of 34.68 PAS 1 responses (SD = 28.45, range 0–92), PAS 2 average 42.88 (SD = 23.44, range 4–81), PAS 3 average 13.72 (SD = 15.35, range 0–63), PAS 4 average 5.52 (SD = 17.07, range 0–82). For the Single unmasked condition there was an average of 81.64 PAS 1 responses (SD = 23.17, range 12 = 100), PAS 2 average 13.2 (SD = 18.03, range 0–76), PAS 3 average 1.44 (SD = 2.27, range 0–8), PAS 4 average 0.4 (SD = 0.2, range 0–1).

A repeated measures ANOVA was conducted on the redundant target conditions split into aware and unaware trials to test the effect of awareness and location on reaction times. This analysis showed an effect of awareness *F*(1,22) = 5.979, *p* = 0.023, *η*_p_^2^ = 0.214, with slower responses for Unaware (Diagonal *M* = 477 ms, SD = 75, Ipsilateral *M* = 498 ms, SD = 92, Contralateral *M* = 497 ms, SD = 84. Averaged across all three configurations: *M* = 485 ms, SD = 80) compared to Aware (Diagonal *M* = 464 ms, *SD* = 68, Ipsilateral *M* = 471 ms, SD = 82, Contralateral *M* = 461 ms, SD = 59. Averaged across all three configurations: *M* = 464 ms, SD = 66) trials. There was no effect of configuration *F*(1.323, 29.109) = 1.481, *p* = 0.24, *η*_p_^2^ = 0.063 (Greenhouse–Geisser corrected, uncorrected dfs = 2, 44) or the interaction between configuration and awareness *F*(1.437, 31.614) = 1.194, *p* = 0.302, *η*_p_^2^ = 0.051) (Greenhouse–Geisser corrected, uncorrected dfs = 2, 44).

**Fig. 4:**
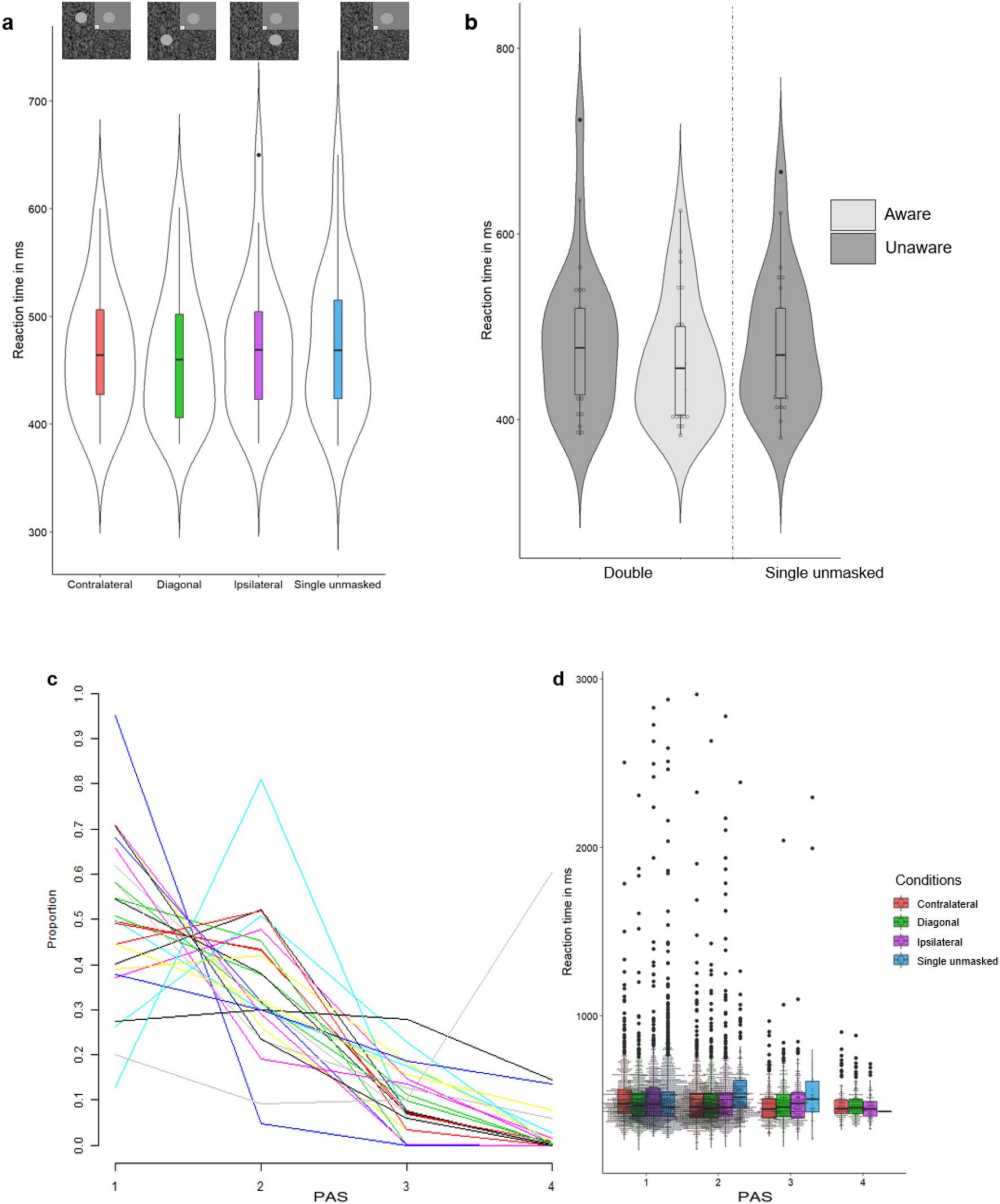
**a** Violin plot with boxplot of the reaction times split by condition. **b** Violin plots with box plots of the mean of the reaction times to the Double Diagonal, Double Ipsilateral, and Double Contralateral condition collapsed together and split by Aware and Unaware trials, and the Single unmasked condition. Each dot represents the mean for each participant. **c** The proportion of PAS 1, 2, 3, and 4 responses per participant. Each participant of experiment 3 is represented by a line. **d** A boxplot of the reaction time per condition and available PAS response with the data by trial of experiment 3 overlaid as a dot plot. **c**, **d** were made in R with the package ggplot2 (Wickham 2016)

The last analysis of experiment 3 was conducted to assess the effect of awareness on the RSE. The three redundant target conditions (Diagonal, Ipsilateral, and Contralateral) were collapsed and then split into Unaware (Diagonal: total *n* = 975, average n per participant = 39, range = 2–88. Ipsilateral: total *n* = 964, average n per participant = 38.56, range 8–87.Contralateral: total *n* = 867, average n per participant = 34.68, range = 0–92) and Aware (Diagonal: total *n* = 1443, average n trials per participant = 57.72, range = 6–97. Ipsilateral: total n = 1447, average n trials per participant 57.88, range 7–88. Contralateral: total *n* = 1553, average n trials per participants 62.12, range 4–100) trials. The Double Aware trials were then compared to the Double Unaware trials and Single unmasked trials (only including Single unmasked trials where participants accurately reported to be unaware of the absent, masked redundant target). This revealed that the Double Aware trials (*M* = 464 ms, SD = 66) were significantly faster than the Single unmasked (*M* = 482 ms, SD = 73, *n* = 2041, average *n* trials per participant = 81.64, range 12–100) [*t*(24) = − 2.091, *p* = 0.047, Cohen’s *d* = 0.259]. Consistent with experiment 2, the Double Unaware trials (*M* = 485 ms, SD = 80) were not significantly different from the Single unmasked trials (Fig. 4).

### Summary of experiment 3

The findings from experiment 3 confirmed the findings from the previous two experiments in that the awareness of masked targets was associated with faster reaction times. But there was no difference in reaction time depending on the spatial configuration of double targets. The findings were invariant to the position of the masked target, that is the stimulus configuration did not have an effect on reported awareness, or changes in reaction times. In all three experiments, reaction times to the double target was slower when participants were unaware of the redundant target, but the difference from single target condition was only statistically significant in experiment 1. We have expanded on this issue in the analysis reported below.

## Combined analysis across three experiments

### Redundancy gain

**Fig. 5:**
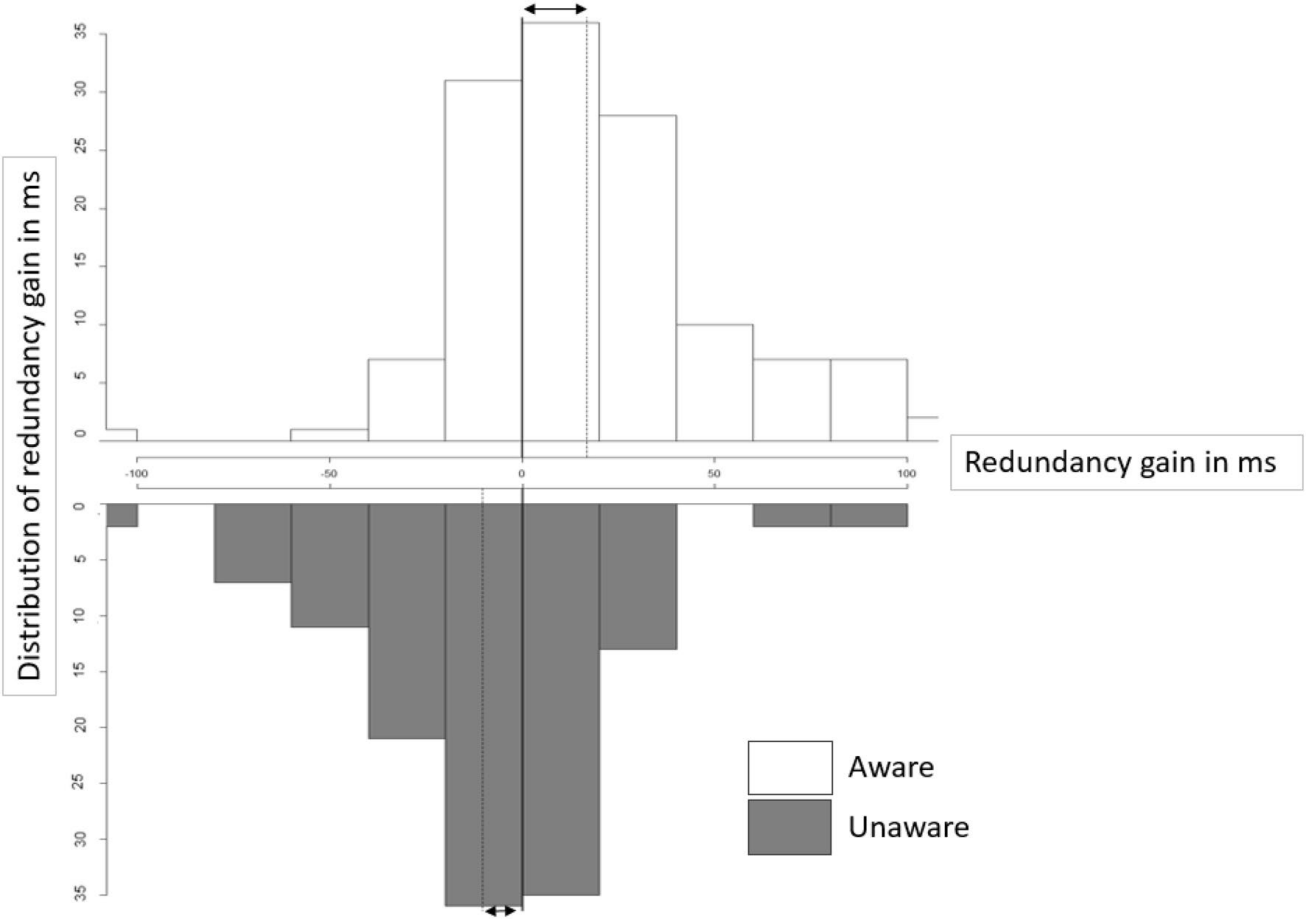
Histogram of redundancy gain ranging from − 100 to 100 ms for all double target conditions from the three experiments. The data has been split into aware and unaware trials and the dotted lines represent the mean for each of the distributions. The solid lines represent the comparison to the Single unmasked conditions. The arrows are included to highlight the difference between the distribution means and the baseline

Redundancy gain is the difference between RTs to a single and a double target presentation. As reported visual awareness of the masked target consistently led to faster RTs across all three experiments, a positive and significant redundancy gain was reported in all three experiments. We also found that when the participant was unaware of the presentation of the masked target, reaction times were numerically slower indicating a negative or low redundancy gain. In Fig. 5 we have plotted the distribution of redundancy gain for all participants and for all experimental conditions.

Overall, the mean redundancy gains for aware trials (*M* = 18 ms, SD = 41) is shifted towards positive numbers and is larger than those for unaware trials unaware (*M* = − 10 ms, SD = 36). As the above data summary is equivalent to an internal meta-analysis, any statistical testing of these differences may be problematic (Ueno et al. 2016). There are, however, some overall characteristics that are worth mentioning. The first observation is that both distributions are wide and span across both negative and positive values indicating large variance in behavioural data. Second, the distributions are not symmetrical with respect their peaks and there is a longer positive tail for aware trials and a longer negative tail for unaware trials, indicating that it is more likely for RTs in double stimulus presentations to be slower for unaware trials and faster for aware trials compared to a Single unmasked stimulus. The distribution showing a tendency for positive redundancy gain in aware trials is consistent with the general findings reported for suprathreshold stimuli. The tendency for negative redundancy gains in unaware trials may be indicative of an inhibitory influence of unconscious redundant stimuli on the responses to seen targets. However, an alternative explanation would be that the negative tendency in unaware trials is simply the consequence of post-hoc classification of the trials in aware and unaware categories. That would also be the case if awareness can modulate reaction times. In order to fully investigate this relationship, the next overall analysis was conducted.

### Effect of awareness on reaction time

In the previous analyses, the ratings from the 4-point awareness scale were collapsed into a dichotomous variable (aware/unaware) but did not account for specific PAS responses. We, therefore, conducted an additional analysis, in which the full range of awareness ratings was utilised to test for an association between awareness responses and reaction time. The aim of the final analysis was to model the predictors of reaction time across the experiments. For this analysis, we used a Linear Mixed Effect Model. The analysis was conducted in R Studio (RStudio Team 2016) using the lmer() function of the lme4 package (Bates et al. 2015). The model was created from the maximal random effects structure method (Barr et al. 2014) as this method has been suggested to improve the chance of models yielding generalizable results. In this analysis we collapsed all the conditions by number of targets. Thus, the single target conditions of the three experiments were grouped as the single target group and the double target conditions were in the double target group as the results of experiments two and three indicated that there were no differences between the double target conditions based on the spatial relationship between the targets. The model was also tested with the conditions entered ungrouped, but this factor was removed from the model. The Single(masked) condition of experiment 1 was excluded from this analysis.

The following is a list of variables which were eliminated from the final model. Experiment number was removed from the model after comparing the model with and without this variable included, which yielded nonsignificant chi-square results. This means that differences between experiments did not serve as a useful predictor of reaction time in the current paradigm. Testing the model with and without the specific conditions showed that the conditions were not predictive of reaction time but that whether the condition contained one or two targets on the other hand was a significant predictor. This finding confirmed the results of experiments 2 and 3 in the lack of differences found in reaction times between the double target conditions.

The final model was as follows: the fixed effects were PAS (estimate = 27.428, standard error = 10.597, *t*-value = 2.588, *p* < 0.01), number of targets (estimate = 33.223, standard error = 7.325, *t*-value = 4.536, *p* < 0.001), and the interaction between PAS and number of targets (estimate = − 24.679, standard error = 5.513, *t*-value = − 4.477, *p* < 0.001).

**Fig. 6:**
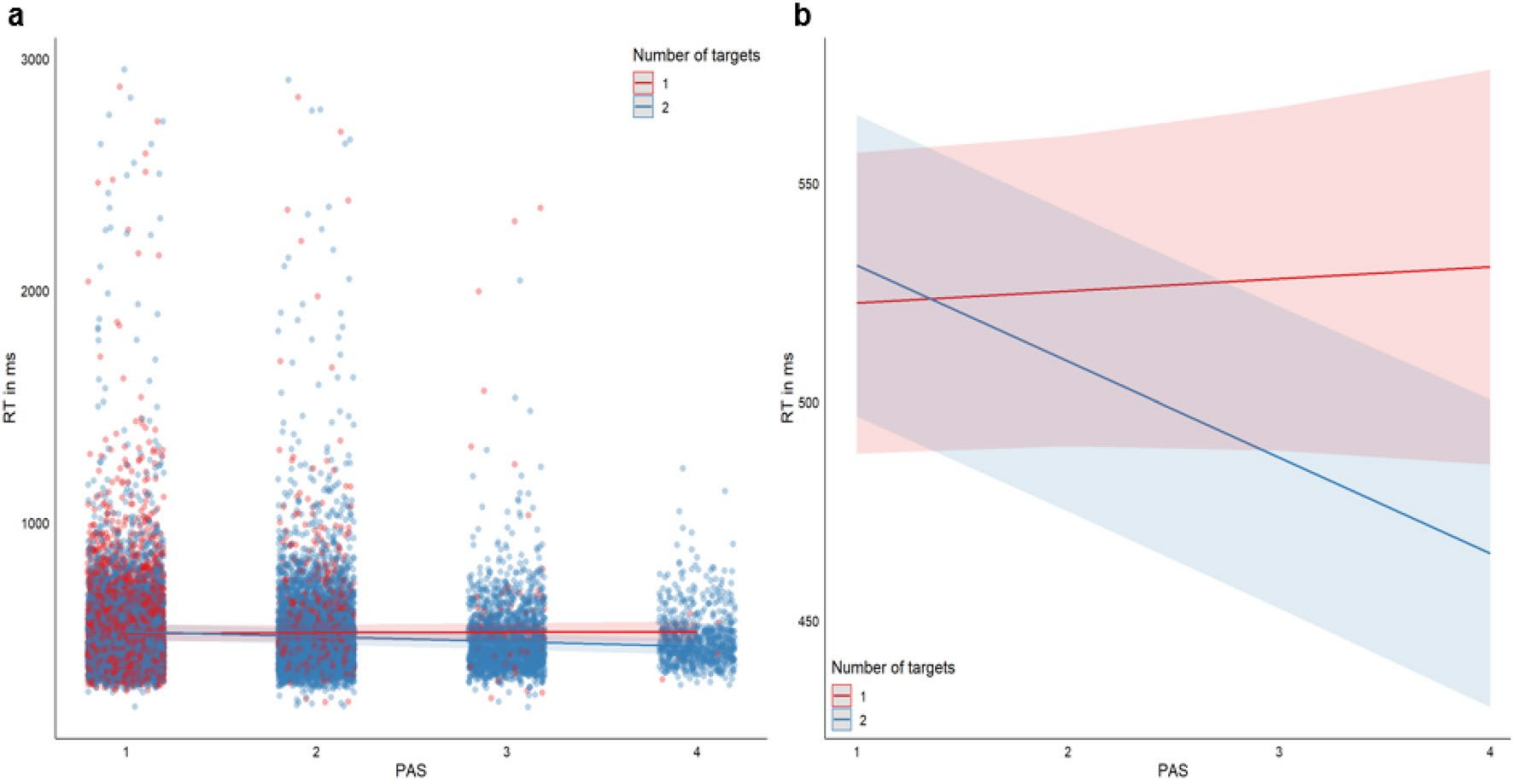
**a** Model plotted with the marginal effects values predicted with the function ggpredict() in the package ggeffects (Lüdecke 2018). The predictions of the model are shown together with the datapoints from all three experiments split by PAS response (1, 2, 3, 4) and the number of targets. **b** The marginal effects of model prediction without the data plotted over the prediction lines. Both figures were made in R with the package ggplot2 (Wickham 2016) as a part of the Tidyverse package (Wickham et al. 2019). The error margins represent the 95% CI as predicted for the model

 The random effects were individual participants which accounted for 23.49% of the variance, block accounted for 2.42% of the variance, and block by experiment accounted for 0.4%. See Tables 1, 2 for the means and standard deviations of the components of the model. The model was fitted with a BOBYQA optimizer (Powell 2009) in the Minqa package (Bates et al. 2014). The general trend of the fixed effects were also the same when analysed alone in a linear model (not accounting for the variance soaked up by the random factors listed above) with PAS (estimate = 118.407, standard error = 11.698, *t*-value = 10.122, *p* < 0.001), number of targets (estimate = 69.669, standard error = 8.023, *t*-value = 8.684, *p* < 0.001), and the interaction between PAS and number of targets (estimate = − 64.546, standard error = 6.067, *t*-value = − 10.639, *p* < 0.001) (Fig. 6).

**Table 1:** Descriptive statistics of the reaction time for the Single and Double conditions collapsed and split by PAS response

	Single	Single PAS1	Single PAS2	Single PAS3	Single PAS4	Double	Double PAS1	Double PAS2	Double PAS3	Double PAS4
Mean	524 ms	515 ms	574 ms	619 ms	469 ms	511 ms	521 ms	510 ms	495 ms	497 ms
SD	190	176	236	391	117	181	197	185	136	114

**Table 2:** Descriptive statistics of the analysis which contributed to the final model. The model comparisons were done with ANOVAs in R

Model		AIC	BIC	Chi-square
Test for additive or interactive relationship between PAS and number of targets	Additive	251,535	251,606	< 0.001
	Interactive	251,517	251,596	
Test for inclusion of experiment as a fixed factor (additive)	Model without	251,516	251,586	0.6687
	Model with	251,517	251,596	
Test for inclusion of block by experiment as a random factor	Model without	251,540	251,603	< 0.001
	Model with	251,516	251,586	
Test for inclusion of condition as a random factor	Model without	251,516	251,579	0.1183
	Model with	251,516	251,586	
Test for inclusion of participants as a random factor	Model without	256,009	256,064	< 0.001
	Model with	251,516	251,579	
Test for inclusion of block as a random factor	Model without	251,523	251,578	< 0.01
	Model with	251,516	251,579

## General discussion

In three experiments we systematically investigated how the magnitude of the RSE was modulated by the level of reported awareness of the masked redundant targets. Across the three experiments we found faster reaction times for multiple targets compared to a single target when participants reported to have some experience of the masked target. We observed larger RSE when participants reported higher levels of awareness of the masked targets. This suggests that for RSE to occur in healthy observers they need to be aware of all targets that are presented. On the other hand, when participants reported to be unaware of a masked visual target that was presented, their responses were slower for double targets compared to the Single unmasked targets, although statistical significance testing led to a mixed picture in individual experiments. However, when the redundancy gain was calculated for the aware and unaware trials of the double target conditions across all three experiments (Fig. 5) most unaware responses yielded either no or a negative redundancy gain. Thus, the presence of a redundant target of which the participant is unaware appears to inhibit the response to a seen target or at least lead to a marked absence of facilitatory redundant target effect.

An interesting observation from the LMEM analysis was that the effect on reaction time associated with the PAS responses diverged for the single compared to the redundant target conditions. In the redundant target conditions the higher PAS responses were associated with faster responses, while the single targets responses showed a slight slowing of reaction time. This was the case both according to the predictions from the marginal effects of the model and the descriptive statistics with reaction times for the single (except for Single PAS 4 response; however, this bin contained a very small amount of data and may not be representative) and double target conditions split by the four step PAS responses (see Table 1 and Fig. 6a, b). The findings from this analysis show that faster reaction times in redundant stimulus condition varies as a function of subjective awareness with enhanced visibility of the redundant targets leading to faster responses. The correlation between conscious experience and faster reaction times is interesting. However, further studies are needed to establish the causes for this relationship. That is, to establish if there is a relationship where conscious experience boosts perceptual processing, decision making, motor planning, execution or whether it is the combination of certain neuronal state that leads to faster processing, that also facilitates the conscious experience.

These findings are somewhat difficult to place in the body of research as most commonly in RSE paradigms, healthy observers’ subjective awareness of targets has not been recorded on a trial by trial basis. However, in the context of manipulating the observer’s experience by using weak or barely detectable targets there are some relevant comparisons to be made. One example is Savazzi and Marzi (2002) who used targets set to a luminance leading to low detection rate (individualised levels) when measuring speed of processing. They found that RSE could be observed for redundant targets presented at detection threshold when they were displayed together with a suprathreshold stimulus. They also found that when they paired a suprathreshold target with a target that was well below the observers’ detection level they did not observe an RSE. When considering their findings together with ours, one possible conclusion is that RSE can be present even if the targets are only barely detectible. However, once the redundant target falls outside of detection (due to internal or external factors) no RSE is observed. In Savazzi and Marzi’s experiment, this was mainly due to external factors (strength of signal of the target). In our experiments this may have been due to internal states of the participant, because the contrast of the targets used in all conditions were kept constant for each participant. Thus, the variation in reaction time that we observed was associated with a subjective state and not to any variation in target signal strength.

Here we have reported that when visual awareness of stimuli was impaired under CFS there was no blindsight-like performance in healthy observers as there was no redundancy gain when the participants reported to be unaware of masked targets. This questions the comparability of findings regarding unconscious vision in patient studies (Celeghin et al. 2015b; Georgy et al. 2016; Leh et al. 2006; Marzi et al. 1996, 1986; Savazzi and Marzi 2002; Tomaiuolo et al. 1997; van Koningsbruggen et al. 2017) and those studies in healthy adults using CFS to simulate unconscious vision. Something that emerges from the body of prior evidence and the findings reported here is that the extent of unconscious processing depends on the methods used to supress the conscious experience. Visual stimuli supressed using backward masking in healthy adults (e.g., Milders et al. 2006) or presentation within neurologically induced visual deficits mentioned above, seem to lead to some processing albeit without conscious awareness. Although some unconscious processing can take place for supressed stimuli under binocular rivalry (Bannerman et al. 2011), CFS induced supression on the other hand appear to, if not eliminate, at least significantly reduce unconscious processing (Moors et al. 2017; Rothkirch and Hesselmann 2018). This leads us to question the viability of CFS as a suitable method to study unconscious vision in healthy observers as a model of blindsight-like performance.

From the findings of experiments reported here, we show that when perceptual awareness is reported on a trial by trial basis, there is a relationship between the strength of perceptual awareness and RT such that higher reported awareness is associated with faster responses. There are, however at least two obvious ways of interpreting the basis for this finding. One is that attention fluctuates over time and cannot be maintained at a steady level across the visual field and throughout the duration of an experiment. The reported awareness is a direct result of such fluctuation with increased attentional allocation, leading to higher likelihood that masked stimuli may break into conscious awareness. In this explanation, slower RTs for unaware responses to redundant targets instead of being a sign of inhibition, are simply a direct result of removing faster responses from the total RT distribution (since they were associated with aware response) leaving behind a set of longer RTs. An alternative explanation may be that we have devised a series of experiments where redundant stimuli are at the verge of conscious awareness and many factors including fluctuations in internal noise, arousal/alertness or even state of attention may interact to push the presentation of a given stimulus over the threshold for awareness and it is this subjective conscious awareness that boosts faster orienting behaviour or even more efficient decision making and response execution leading to faster reaction times. Therefore, it is the increased attention that leads to faster responses in the former, and in the latter, it is the fluctuation in awareness that leads to changes in RT. Experiments reported here do not allow for evaluation of these two possibilities. It is likely that these alternatives can be examined in experiments involving objective measures of attention such as EEG (e.g., Helfrich et al. 2018). Such classification may help to elucidate the relationship between awareness and attention.

In conclusion, we measured reaction times to the onset of a seen (unmasked) target whilst the presentation of a masked redundant target with fixed stimulus characteristics may or may not have led to subjective visual awareness of the redundant target on trial by trial basis. We demonstrated that the amplitude of redundancy gain was correlated with reported subjective awareness. The same pattern of findings was observed irrespective of location of the redundant target or its positional uncertainty. The findings were not in line with predictions based on clinical populations where the unconscious processing was due to damage to early cortical regions.

## Data Availability

Anonymised aggregates of the data from the three experiments is available on Open Science Framework. Project title: “Conscious awareness facilitates and unconscious processing inhibits processing speed in the Redundant Signal Effect in Healthy observers” View only link: https://osf.io/ewh95/?view_only=21db6d45a85f49de8e71bc72b9f9f489.

## Code availability

Not applicable. All of the analysis was done in the statistics software SPSS. The Linear Mixed Effect Model analysis was conducted in R.
